# The association between low pre-operative step count and adverse post-operative outcomes in older patients undergoing colorectal cancer surgery

**DOI:** 10.1186/s13741-020-00150-8

**Published:** 2020-07-02

**Authors:** Simon J. G. Richards, Pippa M. Jerram, Christian Brett, Michelle Falloon, Frank A. Frizelle

**Affiliations:** 1 Christchurch Public Hospital, University of Otago, Christchurch, New Zealand; 2 Department of Anaesthesia, Christchurch Public Hospital, University of Otago, Christchurch, New Zealand

**Keywords:** Frail, Pre-operative, Complications, Post-operative outcomes, Step-count

## Abstract

**Background:**

Multiple tools exist estimating perioperative risk. With an ageing surgical demographic, frailty is becoming an increasingly important concept in perioperative medicine due to its association with adverse post-operative outcomes. Reduced physical activity is a hallmark of frailty, and we postulate that a low pre-operative step count may be an objective measure of frailty. This study aimed to determine the association between low pre-operative step count and post-operative outcomes in patients undergoing elective colorectal cancer surgery.

**Methods:**

A prospective analysis of 85 older patients undergoing major elective colorectal surgery was performed at a tertiary centre between October 2017 and October 2018. Patients aged 65 years and over who met inclusion criteria were provided with an activity tracker to wear for 14 days prior to planned surgery. Their median daily step count was measured and a cut-off of < 2500 steps/day was used to define a reduced step count. Primary outcomes included length of stay and 30-day post-operative complication rate. Multivariable logistic regression analyses were used to analyze the influence of low pre-operative step count and other preoperative variables, on post-operative outcomes including mortality, prolonged hospital admission, and complication rates.

**Results:**

Of 85 patients, 17 (20%) were identified as having a low pre-operative step count. A low pre-operative step count was associated with a significantly increased length of stay (14 vs. 6 days, IRR 2.09, 95% CI 1.55–2.83, *p* ≤ 0.01) and rate of major post-operative complications (29.4% vs. 8.8%, OR 3.34, 95% CI 1.03–14.3, *p* = 0.04). It was also associated with significantly increased rates of discharge to care facilities (*p* < 0.01) and requiring support on discharge (*p* = 0.03).

**Conclusion:**

Low pre-operative step count (< 2500 steps/day) is predictive of an increased risk of post-operative morbidity in patients undergoing elective colorectal surgery. Accurate preoperative identification may allow for treatment modification and tailored perioperative care. The possibility of using a wearable activity tracker as a simple but powerful pre-habilitation tool is raised as an important avenue for future study.

**Trial registration:**

Australian New Zealand Clinical Trials Registry (ACTRN12618000045213).

## Introduction

The global population aged 65 years and older currently sits at approximately one billion with a steep upward trajectory predicted in this demographic (World Health Organization 2015). The number of older patients presenting for surgery is increasing at an even greater rate (Partridge et al. 2012). Preoperative risk assessment in this cohort is challenging as age and comorbidity alone are insufficient to capture the physiologic heterogeneity present in this cohort. Commonly used metrics are often based on a single organ system and fail to address the patient’s global functional reserve (Minto and Biccard 2014). Accurate risk assessment is important for three reasons: to facilitate patient-centred decision making, to identify patients suitable for preoperative intervention and optimisation and to guide perioperative care.

Frailty is a concept that is becoming an integral component of perioperative risk assessment in older patients (Amrock and Deiner 2014; Beggs et al. 2015); frailty has been independently associated with an increased risk of perioperative morbidity and mortality, prolonged hospital admissions, and discharge to care facilities (Fagard et al. 2016; Lin et al. 2016). The frailty syndrome is a state of reduced physiologic reserve seen with increasing frequency with advancing age (Chen et al. 2014). It is independent of any organ-specific diagnosis and has a significant functional component (Conroy and Elliott 2017). The frailty phenotype, as described by Fried et al. (Fried et al. 2001), remains fundamental and comprises five key domains: unintentional weight loss, physical weakness, exhaustion/tiredness, slow gait speed and low activity levels. While clearly associated with advancing years, frailty and ageing are not synonymous (Beggs et al. 2015; Xue 2011); in a large prospective community study of cardiovascular risk factors, only 25% of those aged over 85 years were identified as frail and there was significant variation among different geographic, ethnic and socioeconomic groups of similar age (Fried et al. 1998). It is commonly observed that frailty is easy for a clinician to identify, but hard to define (Gobbens et al. 2010). The lack of a universal operational definition and standardised assessment tool has limited quantitative research and left myriad options for identifying frail individuals. Many assessment tools are time-consuming to perform, rely on multiple subjective measures and may not be well suited to the non-research clinical environment (Dent et al. 2016).

Low activity levels and physical slowness are features of the frailty phenotype (Kristjansson et al. 2010), and regular physical exertion has been shown to be protective against the development of frailty (Buckinx et al. 2017; Huisingh-Scheetz and Walston 2017). In the peri-operative setting, low levels of post-operative physical activity have been shown to be associated with adverse outcomes in surgical patients (Tudor-Locke et al. 2011a; Landi et al. 2010). Low preoperative activity levels have been correlated with established markers of perioperative risk including poor performance in cardiopulmonary exercise testing (Daskivich et al. 2019).

We propose that objectively quantifying preoperative physical activity may provide a novel and pragmatic marker of frailty and perioperative risk. By providing a wearable activity tracker to patients preoperatively and using daily step count as a metric, we hypothesise that a low preoperative step count may be associated with adverse postoperative outcomes. By capturing data continuously over multiple days, we aim to produce a more accurate picture of functional reserve in this cohort of older patients than single snapshot assessments common to most frailty assessment tools. The aim of this study was to determine the correlation between low pre-operative step count and post-operative outcomes in patients undergoing resection for colorectal cancer.

## Methods

### Patient population

A prospective analysis of older patients undergoing major elective colorectal surgery between October 2017 and October 2018 was performed. Older patients (defined as aged 65 years or older) were identified and recruited prospectively through surgical outpatient and preadmission clinics. Inclusion criteria were (i) age 65 years or older; (ii) undergoing an elective colorectal abdominal operation within six months; and (iii) ambulatory, including with the use of walking aids. Exclusion criteria were (i) inability to obtain valid consent, (ii) stoma reversal surgery, (iii) non-abdominal procedures, (iv) non-ambulatory patients and (v) non-elective surgery. Patients requiring walking aids were excluded due to the potential to interfere with step count collection.

Following identification and confirmation of eligibility, informed consent was obtained, and patients were enrolled on a prospectively collected, anonymised database. Baseline socio-demographic and clinicopathological data were collected at enrolment.

Postoperative management was not altered by the study and the clinicians caring for the patients during their post-operative stay were not part of the study protocol. Post-operative outcomes assessed included mortality, length of hospital stay, the development of post-operative complications, readmission and discharge to a care facility. Post-operative complications were graded according to the Clavien-Dindo classification (Dindo et al. 2004), with major complications defined as a score of ≥ 3. Patients were followed-up at 30 and 90 days.

### Wearable activity trackers

At the point of recruitment, patients were issued with a Garmin vívofit® 3 (Garmin, Olathe, KS, USA) wrist-worn activity tracker, with a battery life of approximately 1 year, allowing the patients to wear the device continuously without the need for charging. Steps taken, distance travelled and calories burned were monitored by the wearable activity tracker and stored on the device for up to 90 days. Patients were asked to wear their devices continuously, and daily step count was recorded for 14 days pre-operatively. The device was retrieved, and data downloaded on the day of surgery.

### Frailty assessment and determination of step threshold

Pre-operative frailty was assessed using two validated frailty scales, the Edmonton Frail Scale (EFS) (Rolfson et al. 2006) and the modified frailty index (mFI), in addition to assessing patient pre-operative step counts. Patients scoring 8 or more using the EFS or ≥ 0.27 using the mFI were classified as frail.

Based on the described association between frailty and reduced physical activity, it was postulated that this association would be reflected in a reduced daily step count when compared to more robust peers. In a community-based study of activity in older ambulatory adults, Tudor-Locke et al. (Tudor-Locke et al. 2011b) found that 2500 steps per day or fewer was a basal step count seen in the least active individuals. We chose to use 2500 steps/day as a threshold level below which patients were classified as low step count individuals. Multiple authors (Tudor-Locke et al. 2011a; Hewitt et al. 2015) have shown the prevalence of frailty in the surgical population being approximately 20%. Using < 2500 steps/day as a threshold in our cohort resulted in 20% being classified as having a low level of preoperative physical activity. Finally, receiver-operator curve analysis confirmed that the 2500 steps threshold performed well in identifying frail individuals as defined by an EFS score of greater than 7 (sensitivity 0.75, specificity 0.89, AUC 0.93).

### Statistical analysis

The sample size calculation was based on previous literature estimating approximately 20% prevalence of frailty and low levels of physical activity in older adults (Hewitt et al. 2015; Collard et al. 2012). Based on local data that showed a mean post-operative length of hospital stay of 7 days (SD 1.5) amongst this age group, at a power of 90% and two-sided alpha value of 0.05, a minimum sample size of 94 patients will be required to detect what was deemed to be the minimum clinically significant difference of one day for the inpatient duration between the groups. To allow for attrition, 100 patients were recruited.

Continuous variables were reported as medians with interquartile range (IQR) and categorical variables as whole numbers and percentages. Univariable analysis was performed to compare baseline characteristics between low and normal step count patients. This included chi-square tests for categorical variables and Kruskal-Wallis test for non-parametric continuous variables. Univariable and multi-variable adjusted odds ratios with 95% confidence intervals were used to explore variables associated with differences in outcome. Length of stay (LOS) was evaluated as Poisson count data, was determined to be over-dispersed and as such, analysed using negative binomial regression. Incidence rate ratios from these analyses were interpreted as the relative number of days in the hospital when compared the reference (normal-step count) group.

Multivariable logistic regression analysis was performed on all pre-operative variables, including low step count, that were predicted to affect post-operative outcomes using backward stepwise selection based on Akaike information criterion (AIC). Model performance was assessed using Harrell’s concordance index (C-index). Regression coefficients from a multivariable analysis were reported as odds ratios with 95% confidence intervals (95% CI). *P* values < 0.05 were considered statistically significant. All statistical analysis was performed using RStudio software (version 3.3.2; www.rstudio.com).

### Ethics

This study was approved by the New Zealand Health and Disability Ethics Committee reference 17/CEN/170. Locality approval was obtained from the Canterbury District Health Board. Full prospective written informed consent was obtained from all participants. This manuscript adheres to the applicable equator guidelines.

## Results

### Baseline demographic and clinicopathological characteristics

**Fig. 1:**
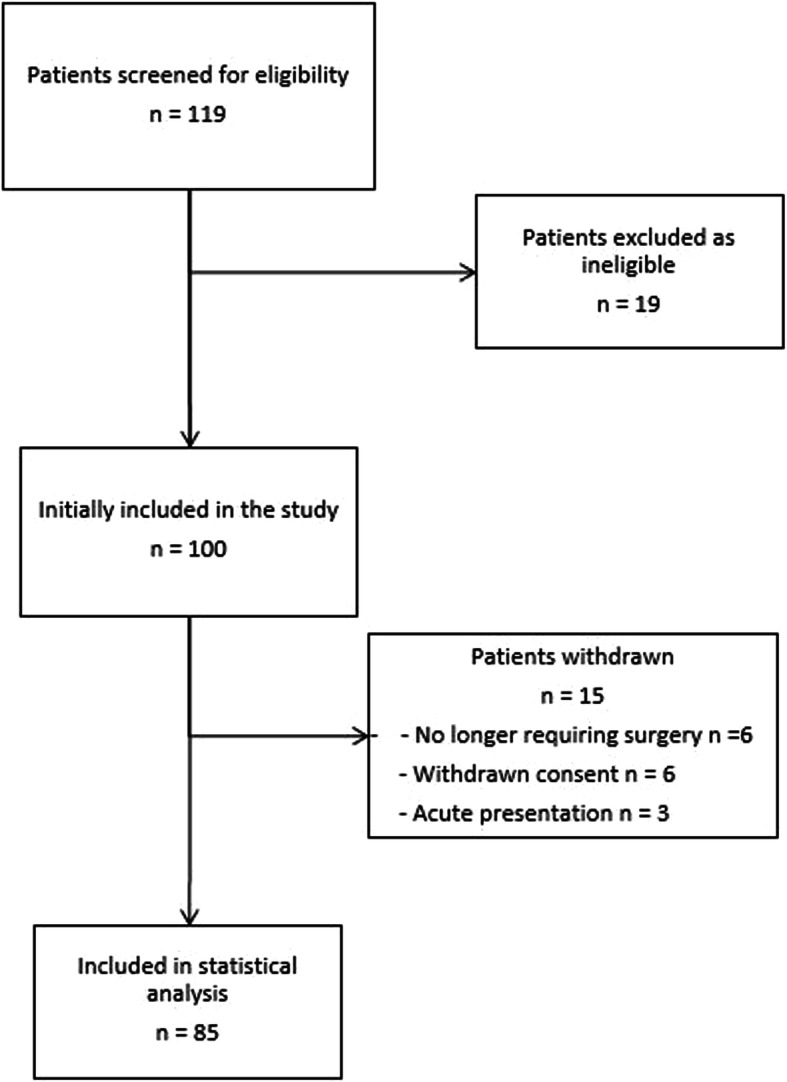
Patient recruitment overview

The patient recruitment process is outlined in Fig. 1. There were 119 patients screened for eligibility. A total of 100 patients were identified that met the inclusion criteria and were recruited into the study. Fifteen patients withdrew from the study. Six no longer required or decided against surgery, 6 withdrew consent and 3 presented acutely before their date of surgery. A total of 85 patients were therefore included in the final analysis. The wearable activity trackers were well tolerated with no patients removing the device during the 14-day period.

**Fig. 2:**
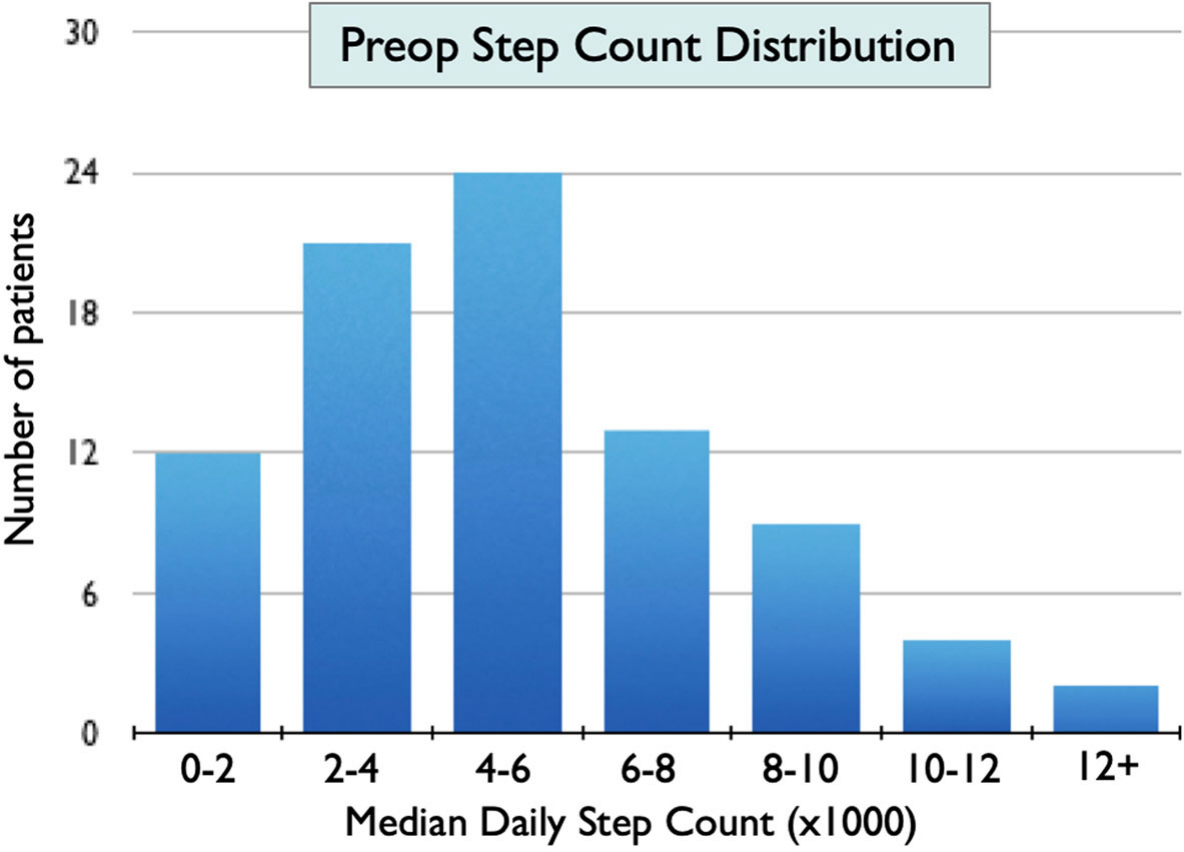
Pre-operative step count distribution with median daily step count assessed over the two weeks prior to surgery

Preoperative steps data are presented in Fig. 2. The median daily step count across all patients was 4569 steps/day (IQR 2727–6830). Of the 85 study patients, 17 (20%) were classified as having a low preoperative step count with a median of < 2500 steps/day. The remainder were classified as having a normal step count.

Baseline demographic, clinicopathological, and treatment variables are shown in Table 1. The median age of the study population was 76 years (IQR 72–81). Patients with a low pre-operative step count had a median age of 78 years (IQR 76–81) compared to 75 years (IQR 70–81) in the normal step count group (*p* = 0.16). The median BMI overall was 27 kg/m^2^ (IQR 25–32), and there was no difference in BMI between the two groups (*p* = 0.79). Low step count patients were overrepresented in ASA grades III or IV with 76.5% of this group being classified ASA III/IV compared to 42.6% of normal step count patients (*p* = 0.06). There was no difference in tumour location between groups (*p* = 0.51); however, low step count patients were more likely to have an open operation with 76.5% having an open procedure, compared to 38.2% of normal step count patients (*p* = 0.01).

**Table 1:** Baseline demographics and clinicopathological characteristics of the study population

Patient variables	All patients (*n* = 85)	Normal step count (> 2500 steps, *n* = 68, 80%)	Low step count (< 2500 steps, *n* = 17, 20%)	Odds ratio (95% CI)	***p*** value
**Age (years)**					
Median (IQR)	76 (72–81)	75 (70–81)	78 (76–81)	1.07 (0.97–1.19)	0.16
**Sex (*n*, %)**					
Female	43 (50.6)	37 (54.4)	6 (35.3)	Ref	
Male	42 (49.4)	31 (45.6)	11 (64.7)	2.55 (0.80–9.06)	0.13
**Ethnicity (*n*, %)**					
NZ European	78 (91.8)	62 (91.2)	16 (94.1)	Ref	
Maori	3 (3.5)	3 (4.4)	0	-	
Other	4 (4.7)	3 (4.4)	1 (5.9)	1.17 (0.05–14.3)	0.91
**BMI kg/m**^**2**^					
Median (IQR)	27.0 (24.5–32.1)	27.3 (24.9–33.0)	26.9 (23.9–30.0)	0.98 (0.89–1.09)	0.79
**BMI categorized (*n*, %)**					
Normal	23 (27.1)	16 (23.5)	7 (41.2)	2.27 (0.72–6.94)	0.15
Underweight	2 (2.4)	2 (2.9)	0	-	-
Overweight	32 (37.6)	28 (41.2)	4 (23.5)	0.44 (0.11–1.39)	0.19
Obese	28 (32.9)	22 (32.4)	6 (35.3)	1.14 (0.35–3.41)	0.82
**ASA (*n*, %)**					
1–2	43 (50.6)	39 (57.4)	4 (23.5)	Ref	
3–4	42 (49.4)	29 (42.6)	13 (76.5)	3.54 (0.99–14.9)	0.06
**Tumour location (*n*, %)**					
Colon	62 (72.9)	49 (72.1)	13 (76.5)	Ref	
Rectum	23 (27.1)	19 (27.9)	4 (23.5)	0.63 (0.15–2.32)	0.51
**Access (*n*, %)**					
Laparoscopic	46 (54.1)	42 (61.8)	4 (23.5)	**Ref**	
Open	39 (45.9)	26 (38.2)	13 (76.5)	**5.20 (1.57–21.0)**	**0.01**
**AJCC stage (*n*, %)**					
I–II	61 (71.8)	50 (73.5)	11 (64.7)	Ref	
III–IV	24 (28.2)	18 (26.5)	6 (35.3)	1.25 (0.69–2.28)	0.46
**Neoadjuvant treatment (*n*, %)**					
None	69 (81.2)	57 (83.8)	12 (70.6)	Ref.	
Short course	6 (7.1)	4 (5.9)	2 (11.8)	2.38 (0.31–13.7)	0.35
Long course	10 (11.7)	7 (10.3)	3 (17.6)	2.04 (0.40–8.56)	0.35
**Modified frailty index (*n*, %)**					
< 0.27	45 (52.9)	42 (61.8)	3 (17.6)	**Ref**	
≥ 0.27	40 (47.1)	26 (38.2)	14 (82.4)	**7.15 (1.75–39.0)**	**0.01**
**EFS (*n*, %)**					
Not frail (≤ 7)	73 (85.9)	65 (95.6)	8 (47.1)	**Ref**	
Frail (≥ 8)	12 (14.1)	3 (4.4)	9 (52.9)	**19.0 (3.68–130)**	**< 0.01**
**Living status (*n*, %)**					
Home	83 (97.6)	68 (100.0)	15 (88.2)		
Care facility	2 (2.4)	0	2 (11.8)	-	**-**

Patients with a median daily pre-operative step count with < 2500 step/day were classified as having a low step count

The Edmonton Frail Scale (EFS) classified 12/85 (14%) patients as frail while the modified frailty index (mFI) found 40/85 (47%) to be frail. Of the 17 patients classified as having a low preoperative step count, 9 (53%) were classified as frail based on the EFS and 14 (82%) based on the mFI. Of those classified as having a normal pre-operative step count, 3 (4 %) were classified as frail based on the EFS and 26 (38%) based on the mFI.

### Post-operative outcomes

**Fig. 3:**
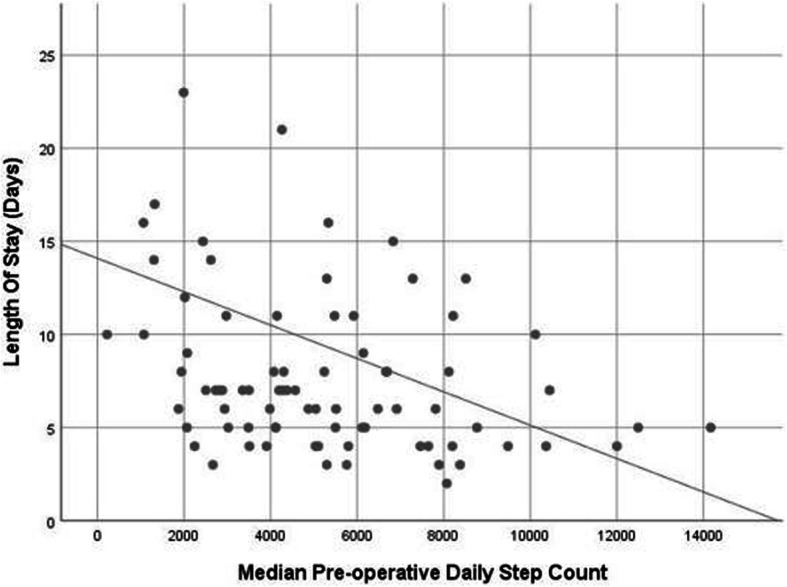
Correlation between median daily pre-operative step count and post-operative length of hospital stay. Pearson’s correlation coefficient, *r*^2^ = 0.338, and *p* = 0.02

Table 2 summarises post-operative outcomes and complications. The overall median length of stay was 7 days (IQR 5–11). Low step count patients had more than double the length of stay when compared with normal step count patients, staying a median of 14 days (IQR 9–17) and 6 days (IQR 4.8–8), respectively. Low step count patients had an incidence rate ratio of 2.75 (2.19–3.47), (*p* < 0.01) when compared to normal step count patients. After adjusting for preoperative clinicopathological variables, low step count remained a significant independent predictor of prolonged length of stay with an IRR = 2.09 (1.55–2.83), (*p* < 0.01). The correlation between raw pre-operative step count and post-operative length of stay is shown in Fig. 3.

**Table 2:** Comparison of post-operative outcomes between low step count and normal step count patients

Patient variables	All patients (*n* = 85)	Normal step count (> 2500 steps, *n* = 68)	Low step count (< 2500 steps, *n* = 17)	Univariable OR (95%CI)	***p*** value	Adjusted OR* (95% CI)	Adjusted ***p*** value
**Length of stay (days)**							
Median (IQR)	7 (5–11)	6 (4.8–8)	14 (9–23)	**IRR 2.75 (2.19–3.47)**	**< 0.01**	**IRR 2.09 (1.55–2.83)**	**< 0.01**
**ICU/HDU length of stay (days)**							
Median (IQR)	0 (0–1)	0 (0–1)	2 (1–2)	**IRR 5.75 (3.13–11.6)**	**< 0.01**	**IRR 3.14 (1.64–6.05)**	**< 0.01**
**Days in hospital at 90 days (days)**							
Median (IQR)	7 (5–13)	7 (5–11)	17 (9–31)	**IRR 2.39 (1.76–3.24)**	**< 0.01**	**IRR 2.47 (1.71–3.63)**	**< 0.01**
**Clavien-Dindo complication (*n*, %)**							
No	38 (44.7)	34 (50.0)	4 (23.5)	**Ref**		Ref	
Any	47 (55.3)	34 (50.0)	13 (76.5)	**3.25 (1.1–12.1)**	**0.05**	2.02 (0.58–8.23)	0.29
I–II	36 (42.4)	28 (41.2)	8 (47.1)	1.27 (0.43–3.72)	0.66	1.07 (0.35–3.25)	0.90
III–V	11 (12.9)	6 (8.8)	5 (29.4)	**4.31 (1.09–16.7)**	**0.03**	**3.34 (1.03–14.3)**	**0.04**
**Length of stay > 7 days (*n*, %)**							
No	49 (57.6)	46 (67.6)	3 (17.6)	**Ref**		**Ref**	
Yes	36 (42.3)	22 (32.4)	14 (82.4)	**8.04 (2.24–38.6)**	**< 0.01**	**9.76 (2.84–45.6)**	**< 0.01**
**Unplanned readmission at 90 days (*n*, %)**							
No	69 (81.2)	56 (82.4)	13 (76.5)	Ref		Ref	
Yes	16 (18.8)	12 (17.6)	4 (23.5)	1.44 (0.36–4.92)	0.58	1.99 (0.44–8.45)	0.35
**Discharge to rehab/care facility (*n*, %)**							
No	73 (85.9)	64 (94.1)	9 (52.9)	**Ref**		**Ref**	
Yes	12 (14.1)	4 (5.9)	8 (47.1)	**14.2 (3.74–63.2)**	**< 0.01**	**10.4 (2.53–49.2)**	**< 0.01**
**Support on discharge (*n*, %)**							
No	69 (81.2)	60 (88.2)	9 (52.9)	**Ref**		**Ref**	
Yes	16 (18.8)	8 (11.8)	8 (47.1)	**6.67 (2.01–23.0)**	**< 0.01**	**5.33 (1.35–22.8)**	**0.03**
**Post-operative day 90 living status (*n*, %)**							
Home independent	72 (84.7)	61 (89.7)	11 (64.7)	**0.27 (0.09–0.84)**	**0.04**	**0.21 (0.05–0.76)**	**0.02**
Home with care	4 (4.7)	2 (2.9)	2 (11.8)	2.22 (0.08–24.5)	0.60	2.06 (0.09–22.9)	0.56
Hospital inpatient	1 (1.2)	0	1 (5.9)			-	-
N/A	8 (9.4)	5 (7.4)	3 (17.6)			-	-

*IRR* incidence rate ratio

*Adjusted for age, gender, tumour stage and operative access

There was just one death within 90 days amongst the normal step count group. Post-operative complications occurred in 47 (55.3%) patients within 30 days, and 11/85 (12.9%) were major complications (Clavien-Dindo III–V). Low step count patients were more likely to suffer from any complication with 13/17 (76.5%) patients from this cohort suffering from a complication compared to 34/68 (50.0%) normal step count patients (adjusted OR 2.02, 95% CI 0.58 – 8.23, *p* = 0.29). They had significantly more major post-operative complications; with 5/17 (29%) patients having a Clavien-Dindo III-V complication compared to 6/68 (9%) patients with a normal step count (*p* = 0.04).

Multivariable analysis was conducted using backward stepwise selection to assess the association between pre-operative patient factors and the development of post-operative complications. All pre-operative demographic factors, including the presence of a low pre-operative step count, were included in the model A low pre-operative step count remained a significant independent predictor of developing a post-operative complication (*p* = 0.04).

Low step count patients had a higher rate of readmission at 90 days with a readmission rate of 23.5% compared to 17.6% in the normal step count group; however, this difference was not statistically significant. Low step count patients were significantly more likely to be discharged to rehabilitation or a care facility (adjusted OR 10.4, 95% CI 2.53–49.2, *p* < 0.01) and were more likely to require additional support on discharge (adjusted OR 5.33, 95% CI 1.35–22.8, *p* = 0.04).

## Discussion

In this study, we have demonstrated that a preoperative step count of less than 2500 steps per day was associated with more than double the length of hospital stay following colorectal surgery when compared to more active individuals. Preoperative low step count was also shown to be an independent predictor of serious complications and discharge to rehabilitation or other supported care facilities.

Independent older adults in developed countries have been found to take on average 4500 steps per day (Tudor-Locke et al. 2011a) which is remarkably similar to the median daily step count of 4569 found in our study. This implies a degree of generalizability from this group of colorectal cancer patients to a wider population. The same community-based data also informed our threshold of 2500 steps as defining those with a basal level of physical activity; we postulated that significantly frail individuals will fall into this activity category. Supporting this, post hoc receiver-operator analysis of steps vs frailty as identified by EFS gave a sensitivity of 0.75, a specificity of 0.93 and an area under the curve of 0.93 at a cutoff of 2472 steps.

**Fig. 4:**
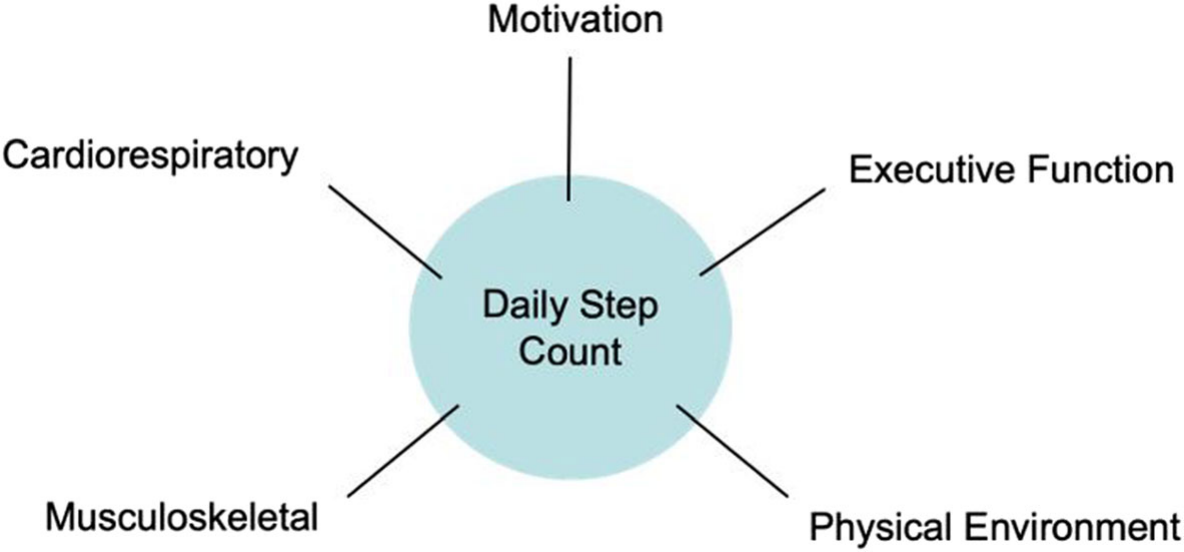
Factors influencing daily step count

Multiple tools exist to identify frail patients each with advantages and disadvantages in the context of a clinical setting. The use of daily step count as a marker of frailty and surgical risk falls into the category of surrogate measures along with grip strength and timed up-and-go. As such, it has the advantage of being truly objective and relatively simple to administer. We propose it may give significantly more insight into a patient’s functional status than other surrogate tools as it is more than a “single snapshot” of an isolated function. As well as providing continuous data over multiple days instead of a few seconds, step count is influenced by a number of factors relevant to the frailty phenotype. This concept is illustrated in Fig. 4.

While step count can now be added as another tool used to stratify surgical risk, it also holds a significant appeal as a tool for preoperative optimization. Prehabilitation is increasingly considered an essential part of the perioperative service, as increasing numbers of older and high-risk patients are considered for major surgery. There has been growing interest in the use of multimodal prehabilitation, with the first randomized trial of its use in colorectal surgery now recruiting (van Rooijen et al. 2019). It is possible that a simple targeted improvement in step count may also be effective. Anecdotally, many patients in our study population reported that wearing the activity tracker was intrinsically motivating even at its most basic setting of displaying a daily step count. There was evidence of some training effect with patients independently choosing to try and improve their daily totals. Considering the multiple factors influencing daily step count (Fig. 4), providing a tailored preoperative target may be able to provide a degree of multimodal prehabilitation with a single intervention.

A strength of this study is that it is the first published investigating the use of wearable activity trackers in pre-operative risk assessment. Previous publications have demonstrated limited applications in the post-operative period (Wolk et al. 2018; Takahashi et al. 2015), but this current publication is the first looking at their use in pre-operative risk assessment.

Several limitations should be considered when interpreting data from this study. Data were collected exclusively in elective colorectal cancer patients and therefore cannot necessarily be extrapolated to other major surgery groups. Despite this, the multi-dimensional nature of step count and quantitative similarity to larger community data is encouraging for generalizability. Data were derived from a single institutional experience with a relatively small group of treating clinicians and a relatively homogeneous patient population, which may limit the application to other settings or ethnicities. Significantly more patients with a low pre-operative step count underwent open as opposed to laparoscopic surgical resection. This surgical decision was made by treating teams independent of any knowledge of step count or formal frailty assessment undertaken as part of this study. While all the reported outcomes associated with low step count were adjusted for surgical access, it remains a potential confounder. Deciding between open vs. laparoscopic access in colorectal surgery is multifaceted, and clinicians will be aiming to balance perceived recovery benefits with a prolonged intra-operative phase. While it is generally accepted that one of the benefits of laparoscopic access in colorectal resection is reduced the length of stay, this has not been able to be demonstrated in study populations aged 65 years and older (Fujii et al. 2016). Finally, pedometer accuracy has been shown to be compromised by slow walking speed (Cyarto et al. 2004), and it may be that step count is underestimated in those frail patients with a slow walking speed.

This study provides another data point emphasising the importance of maintaining physical activity as we age; multiple factors influence this ability and for our frailer patients, it may require a multidisciplinary effort to facilitate as much movement as possible in the perioperative period.

## Data Availability

The datasets used and/or analysed during the current study are available from the corresponding author on reasonable request.

## Abbreviations

AIK: Akaike information criterion
CI: Confidence interval
EFS: Edmonton frail scale
IQR: Interquartile range
IRR: Incidence rate ratio
mFI: Modified Frailty Index
OR: Odds ratio
